# Nutraceuticals against Oxidative Stress in Autoimmune Disorders

**DOI:** 10.3390/antiox10020261

**Published:** 2021-02-08

**Authors:** Carmen Mannucci, Marco Casciaro, Emanuela Elisa Sorbara, Fabrizio Calapai, Eleonora Di Salvo, Giovanni Pioggia, Michele Navarra, Gioacchino Calapai, Sebastiano Gangemi

**Affiliations:** 1 Department of Biomedical and Dental Sciences and Morphofunctional Imaging, University of Messina, 98125 Messina, Italy; carmen.mannucci@unime.it (C.M.); emanuela.sorbara@hotmail.it (E.E.S.); gioacchino.calapai@unime.it (G.C.); 2 Department of Clinical and Experimental Medicine, Unit and School of Allergy and Clinical Immunology, University of Messina, 98125 Messina, Italy; gangemis@unime.it; 3 Department of Chemical, Biological, Pharmaceutical and Environmental Sciences, University of Messina, 98168 Messina, Italy; f.calapai@gmail.com (F.C.); michele.navarra@unime.it (M.N.); 4 Department of Veterinary Sciences, University of Messina, 98168 Messina, Italy; edisalvo@unime.it; 5 Institute for Biomedical Research and Innovation (IRIB), National Research Council of Italy (CNR), 98164 Messina, Italy; giovanni.pioggia@cnr.it

**Keywords:** nutraceuticals, oxidative stress, immunity, autoimmune, ROS, supplement, inflammation, integrators, diabetes, exogenous antioxidants

## Abstract

Antioxidant mechanisms are constituted of enzymes, endogenous, and non-enzymatic, exogenous, which have the role of counterbalancing oxidative stress. Intake of these compounds occurs in the diet. Vegetables, plants, and fruits contain a wide range of alkaloids, polyphenols, and terpenoids which are called “phytochemicals”. Most of these substances are responsible for the positive properties of fruits and vegetables, which are an essential part of a healthy life with roles in ameliorating chronic illnesses and favoring longevity. Nutraceuticals are substances contained in a food or fragment of it influencing health with positive effects on health helping in precenting or treating disorders. We conducted a review illustrating the principal applications of nutraceuticals in autoimmune disorders. Literature reported several studies about exogenous dietary antioxidant supplementation in diverse autoimmune diseases such as rheumatoid arthritis, lupus, diabetes, and multiple sclerosis. In these pathologies, promising results were obtained in some cases. Positive outcomes were generally associated with a reduction of oxidative stress parameters and a boost to antioxidant systems, and sometimes with anti-inflammatory effects. The administration of exogenous substances through food derivates or dietary supplements following scientific standardization was demonstrated to be effective. Further bias-free and extended studies should be conducted that include ever-increasing oxidative stress biomarkers.

## 1. Introduction

Reactive oxygen species (ROS) represent a class of molecules capable of damaging DNA. They are almost always present in the human body since they are produced by cellular metabolism in response to toxic factors or after the intervention of external phenomena. Superoxide anion radicals (O_2_^−^), hydrogen peroxide (H_2_O_2_), hydroxyl radicals (OH), and singlet oxygen (O_2_) are only some of the chemical products belonging to the ROS family [1].

ROS can be augmented by multiple factors and/or due to impaired antioxidant defenses. The loss of redox homeostasis generates stressed cells which in turn produce damage-associated molecular patterns (DAMPs) or autoantigens that could initiate innate immunity and adaptive immunity. This series of events leads to the dysfunction and death of cells via an inflammatory cascade. Oxidative stress and autoimmunity with genetic susceptibility are associated with the pathogenesis of several autoimmune diseases; however, precisely how these two pathways integrate one with each other is not fully understood [2,3] The etiology of autoimmune diseases is many due to genetic and environmental factors. Their prevalence is between the 0.33% and 2%. Disease severity and symptoms like pain are intimately connected to inflammation and oxidative stress. Innate immunity appears to be one main actor in autoimmunity which causes disease progression. Patients suffering from several immune-related diseases such as rheumatoid arthritis (RA), systemic lupus erythematosus (SLE), multiple sclerosis (MS), and diabetes mellitus (DM) type 1 have an altered Type 1 helper (Th1)/Type 2 helper (Th2) activity ratio, augmented Type 17 helper (Th17) responses, as well as Type 0 helper (Th0) and Th1 cytokines serum levels [4].

Antioxidant mechanisms are constituted of enzymes which counterbalance ROS effects. The most studied are superoxide dismutase (SOD), catalase (CAT), glutathione peroxidase (GPx), and myeloperoxidase (MPO). The aim of these mechanisms is the transformation of radicals into less harmful molecules. There are also non-enzymatic and exogenous antioxidants which counterbalance oxidative stress [5,6]. Intake of these compounds occurs in the diet and includes E and C vitamins, carotenoids, and flavonoids [7]. Vegetables, plants, and fruits contain a wide range of alkaloids, polyphenols, and terpenoids which are called “phytochemicals”. Most of these substances are responsible for the positive properties of fruits and vegetables, which are an essential part of a healthy life with roles in ameliorating chronic illnesses and favoring longevity [8]. The Mediterranean diet, rich in these nutrients, is associated with a reduced incidence of chronic diseases [9]. Moreover, although phytochemicals can generate hormetic positive effects on aging and on chronic illnesses, various botanical formulations have been characterized for their citoxicity and apoptosis-inducing activity [10]. 

Exogenous antioxidants could be introduced in the human body through several compounds, either natural or artificial. However, their efficacy has not completely been demonstrated in relation to immune system alterations. In this review, we focused on the latest evidence of the ability of nutraceuticals to influence the formation of ROS in autoimmune diseases.

The term ‘nutraceutical’ was invented in 1989 by Stephen L. Defelice, who established The Foundation for Innovation in Medicine in 1976. There is no internationally recognized definition of a nutraceutical, and various confusing and contradictory definitions have appeared. 

Nutraceuticals are substances contained in a food that have positive effects on health and help in preventing or treating disorders [11]. Their positive effect in many health problems, such as cancer, inflammation, hypertension, cardiovascular diseases, atherosclerosis, obesity, and diabetes, has been previously shown [12]. Moreover, several clinical studies on nutraceuticals have been carried out and results support the effectiveness of nutraceuticals as well as their general safety [13].

Nutraceuticals are also defined by European Nutraceutical Association as ‘nutritional products which have effects that are relevant to health which are not synthetic substances or chemical compounds formulated for specific indications containing nutrients partly in concentrated form [14]. ENA declares that also the terms dietary supplements, dietetic and functional foods are referred as nutraceuticals and they form a common category [15].

In the light of the above-mentioned concepts, we wrote a narrative review illustrating the applications of principal nutraceuticals in autoimmune disorders. 

Electronic databases PubMed, Scopus, and ScienceDirect were used. In this review, we collected and discussed scientific articles published in peer-reviewed journals written in the English language until December 2020 describing the use of nutraceutical products in autoimmune disorders. On the basis of bibliographic research, we commented those nutraceuticals used for autoimmune disorders on which the strongest evidence is present in the scientific literature: exogenous antioxidants (vitamins, flavonoids, coenzymes Q9 and Q10), probiotics, medicinal plants (*Allium sativum*, *Ricinus communis*, *Origanum vulgare, Curcuma longa*), lichen species, melatonin, arginine, omega-3, minerals (selenium, zinc).

## 2. Exogenous Antioxidants

Exogenous antioxidants are fundamental in counterbalancing oxidative stress. These compounds intake happen thanks to diet and include some of the principles listed above. The chemical structure of the principal exogenous antioxidants was represented in a Supplementary Figure S1.

### 2.1. Vitamins

Vitamin E is an effective antioxidant, having 8 isoforms. Vitamin E is co-localized in the mitochondrial membrane and works as peroxyl radical scavenger. Research demonstrated the presence of doubled α-tocopherol concentration in non-asthmatics compared to asthmatic subjects [16]. 

Vitamin C exert its action in collaboration with vitamin E. It eliminates excessive radicals and restores vitamin E to its original form [17]. 

Carotenoids include β-carotene, introduced with the diet (i.e., carrots, lettuce, fruit). Once in the body, it is transformed in retinol (fundamental for eye physiology). It protects against the singlet oxygen [18]. 

Vitamin D is a secosteroid hormone. Its D3 form (cholecalciferol) derives from the skin after intervention of ultraviolet B-light. Its levels are determined mainly from this pathway than from the diet [19]. It has strong healthy effects by acting through the its nuclear receptor in several diseases: osteoporosis, cancer, and autoimmune diseases [20,21]. Vitamin D effects are mainly due to its immune balancing ability, antioxidant and anti-fibrotic activities [19]. Low levels of vitamin D is considered a key factor inducing autoimmune diseases. It has been observed that its supplementation prevents and ameliorates these pathologies [22].

### 2.2. Flavonoids

Flavonoids are typical components of plants (such as green tea leaves and grapes). They are polyphenolic substances constituted by 3 rings: 2 aromatics, A and B and a heterocyclic one, C. Some of them in alternative to the C ring could have an open chain of three carbon atoms. The oxidation and degree of unsaturation of ring C determines the subclass: flavonols and flavones, flavanols, (or catechins), flavanones, anthocyanins, and isoflavones [23].

They have protective effects in autoimmune diseases [24]. Flavonoids exert the renowned beneficial effect in human by targeting multiple cell systems. Nitric oxide (NO) and reactive oxygen species (ROS) are some of their targets thanks to their ability to ameliorate antioxidant enzymes, like inducible nitric oxide synthase (iNOS) and cyclooxygenases (COXs) [25,26].

### 2.3. Coenzyme Q9 and Q10 (CoQ)

Coenzyme Q (CoQ) is arousing lot of interest about its actions as antioxidant in the fight against ROS. Coenzyme Q10 (ubiquinone) is an electron carrier and has a function as protein translocator. Once transformed in ubiquinol it regenerate vitamin E acting as a key player as antioxidant [27,28,29]. CoQ homologues have a different number due to their isoprenoid units (Qn) bind at the 6-position on the benzoquinone ring of the coenzyme Q moiety. In the human body, Q9 and Q10 are present in several organs such as kidney, brain, liver, heart, blood, and cutis [30]. Intracellularly, the different organelles have different concentrations of CoQ. Diabetic patients have an increased vulnerability to oxidative stress due to a minor concentration of the Q9 and Q10 coenzymes in the mitochondria. They showed protective effects after ischemic events [31].

## 3. Probiotics

Probiotics administration was demonstrated having positive effects on several immune related diseases. Among these, for sure rheumatoid arthritis, ulcerative colitis, multiple sclerosis, and hepatic encephalopathy were certainly influenced [32]. Considering different phyla, *Lactobacilli* and *Bifidobacteria* were effective in the balance between Treg and Th17 action. Moreover, in some lung diseases like asthma and COPD probiotics administration ameliorated oxidative stress status [32,33,34]. Literature data suggested the existence of a bidirectional axis between gut microbiota and some organs (i.e., lung, brain). In this scenario, *Lactobacilli* and *Bifidobacteria* are the main actors in reducing chronic inflammation mediators [35].

## 4. Medicinal Plants

Remedies based on plant extracts were used for centuries in folk medicine. During the last decades, modern science focused on the identification of bioactive plant chemical compounds able to produce effects influencing positively human physiology. In fact, some of the most fundamental treatments directly derives from natural components. Individuating potential plant-based drugs aroused a lot of interest in last years and it became a challenge for many researchers [36]. Among plants showing effects on immune system, we chose six of the most investigated. 

### 4.1. Allium Sativum

*Allium sativum*, commonly known as garlic, was reported as having several beneficial effects. It is well tolerated even though it could be detrimental for the stomach when taken at high doses. It has disinfectant properties acting on bacteria, fungus, and parasites. It was demonstrated as having metabolic effects also, ameliorating glucose absorption, and diminishing cholesterol. Anti-neoplastic actions were reported. Its cooked and waited extracts and oils might offer an improved antioxidant effect versus fresh garlic [37,38,39].

### 4.2. Ricinus Communis

*Ricinus communis* L. *(Euphorbiaceae)* is arousing a lot of interest as an anti-neoplastic agent. It is famous among common people as castor plant and it was used in traditional Indian medicine for [40]. Other data highlighted its effectiveness against fungi and pests [41].

### 4.3. Origanum Vulgare

Distributed worldwide, *Origanum vulgare* (Lamiaceae) is used almost everywhere as a spice. It is also a component of traditional medicine for common pathologies like respiratory illness and intestinal diseases [42]. Antimicrobial and antioxidant abilities were also attributed to this plant. Probably, its elevated concentration of volatile oils gives to the plant its antimicrobial potential [43]. It has phenolic compounds including flavonoids and phenolic acids, with a strong antioxidant activity.

### 4.4. Curcuma Longa (Curcumin)

Curcumin is a polyphenolic substance contained in the rhizome of *Curcuma longa*. It is employed as analgesic and wound-repairing agent. Recently findings carried out in humans and laboratory animals reported that curcumin improves autoimmune diseases such as rheumatoid arthritis, multiple sclerosis, psoriasis, and inflammatory bowel disease. Curcumin protective activity in these diseases seems to be due to modulation of inflammatory cytokines and associated signaling pathways in immune cells [44].

## 5. Lichen Species

Lichens also belong to traditional medicine heritage. The products of transformation generated by lichen are the reason of this therapeutic potential. Among these, phenolic molecules are some of these secondary metabolites and the best tested. These metabolites can be categorized in: depsides, depsidones, dibenzofurans, and pulvinic acid derivatives. They demonstrated antioxidants abilities by boosting endogenous enzymatic defenses and non-enzymatic mechanisms [45].

## 6. Melatonin

It is a hormone produced by the pineal gland with a fundamental role in the regulation of the circadian rhythm. During the last decades it has exerted a lot of interest due to its direct antioxidant properties by a scavenger action and by the induction of genes related to antioxidant system activity [46,47].

## 7. Arginine

L-arginine is for humans an essential amino acid with a series of fundamental biological functions during its metabolism. During the last 20 years it emerged its role in ameliorating oxidative stress tolerance and in balancing immunity. The exact antioxidant mechanisms remain still unclear but some hypothesis were conducted on the basis of its potential as a booster of endogenous antioxidant defenses [48].

## 8. Omega-3

In the last years, growing evidence coming from genetic mouse models and clinical studies highlighted the role and underlying mechanisms of ω-3 polyunsaturated fatty acids and their metabolites in the prevention and therapy of autoimmune pathologies such as rheumatoid arthritis, systemic lupus erythematosus, type 1 diabetes, and multiple sclerosis [49].

## 9. Minerals

### 9.1. Selenium

Selenium is an essential micronutrient playing a key role in physiology including regulation of immune activity [50]. Intake of selenium may reduce circulating thyroid autoantibodies in subjects affected by chronic autoimmune thyroiditis [51]. 

### 9.2. Zinc

Zinc is a trace element essential for living organisms with a fundamental role in enzymatic activity and cellular communication, proliferation and differentiation. Zinc has a role also in modulating the immune system. It has been proved the existence of a deficiency of zinc in patients affected by autoimmune diseases [52].

## 10. Autoimmune Diseases

According to the different areas involved in the research, articles were divided and discussed as follows (Table 1 and Table 2):

### 10.1. Rheumatoid Arthritis

Rheumatoid arthritis (RA) is a chronic autoimmune disease characterized by systemic inflammation; patients often present symptoms like swelling, tenderness, and gradual permanent damage to joints. These symptoms lead to functional disability and to an augmented mortality. Unfortunately, RA is pretty common in immune-related disorders affecting from the 0.5% to 1% of the population, occurring 3 times more frequently in women [85]. Its pathogenesis is still an object of study, but T and B cells as well as macrophages and synoviocytes are key players provoking a detrimental cascade based on cytokine release, tissue damage, and ROS generation. Local and general inflammation are natural consequences [86].

Many studies have evaluated the efficacy of alpha-lipoic acid (ALA) in RA patients [53]. In particular, ALA is a potent antioxidant. In this study, ALA did not significantly change the serum levels of pro-inflammatory biomarkers, such as tumor necrosis factor-alpha (TNF-α), interleukin-6 (IL-6), fundamental cytokines implicated in the pathogenesis of RA, C-reactive protein (hs-CRP), and matrix metalloproteinases (MMP-3). The authors did not find a statistically significant correlation with these cytokines and development of AR; therefore, the levels of ROS before the analysis were not significant [53].

One of the last frontiers in terms of supportive therapies is altering gut microbiota. Some authors tested the hypothesis that restoring altered microbiota in RA patients could be useful for controlling cellular damage, oxidative stress, and inflammation. Unfortunately, no beneficial effects of *L. casei* supplementation on the oxidative status of patients with RA were observed [64]. However, some animal studies are in conflict with this result [87] and not all of the results were negative in the human RA research. In fact, probiotic yogurt intake augmented some endogenous antioxidants, although not enough to modify oxidative stress parameters [64].

Sesamin supplements exhibited a protective effect on the modulation of heart and metabolic-related risk factors in women affected by RA. A dose of 200 mg per day for a period of 6 weeks significantly ameliorated some parameters; anthropometric indices, lipid profile, blood pressure, and oxidative stress markers were some of those. Sesamin, which is low-cost and free of side effects, is a valid supplement for the prevention of cardiovascular accidents in RA and in patients with similar risk factors [66].

A study conducted in Iran evaluated the effects of the intake of Coenzyme Q10 (CoQ10) on plasmatic levels of oxidative stress and inflammatory markers such as malondialdehyde (MDA), total antioxidant capacity (TAC), IL-6, and TNF-a in RA patients. According to the results, CoQ10 intake for 8 weeks lead to a massive reduction of blood MDA. Moreover, the capability of CoQ10 of interfering with inflammatory mediators could be due to its efficiency in blocking the nuclear factor kappa B (NF-kB) cascade. Plasmatic TAC and IL-6 were not affected [56].

Fish oil, concentrated fish oil, and their combination with evening primrose oil were tested for their antioxidant activity in a 3 month study of patients affected by RA. The concentrated fish oil group displayed augmented levels of thiobarbituric acid reactive substances (TBARS) and nitrogen dioxide (NO_2_^−)^ in blood; in addition, GSH levels were augmented as well, with a reduction of plasmatic H_2_O_2_. Similar results were achieved using the concentrated fish oil combined with evening primrose oil. In addition, the last group had increased activity of SOD. These data confirmed the speculation of the authors regarding the antioxidant capacities of the described oils by ameliorating the endogenous antioxidant enzymes [60].

### 10.2. Systemic Lupus Erythematosus

Another important autoimmune disease is systemic lupus erythematosus (SLE). Its importance is due to its high mortality rate. SLE pathogenesis is still unclear. Genetics, environment, drugs, pregnancy, and infection are only part of the complex disease mosaic [88]. It is a multiorgan disorder: mucosa, joints, kidneys, lungs, hearts, and particularly nerve cells in the peripheral nervous system are particularly targeted [89]. Among the risk factors for SLE is oxidative stress. Due to its severity, studies have been conducted that evaluated the potential use of antioxidant drugs and integrators as treatments. SLE subjects were tested using either a placebo or vitamins C and E (500 mg vitamin C and 800 IU vitamin E daily) for 3 months. Oxidative stress was evaluated by dosing with malondialdehyde (MDA) and allantoin. Erythrocyte superoxide dismutase, glutathione peroxidase, and plasma total antioxidant power (as FRAP value) were the parameters considered for monitoring antioxidant capacities. 

As a result, the treated group had an increase in ascorbic acid and alpha-tocopherol as well as a substantial decline of MDA. Lipid peroxidation diminished without interfering with endothelial function [68]. As demonstrated for RA, fish oil also showed its efficacy in SLE. Pro-inflammatory cytokines such as IFN-alpha, IFN-gamma, IL-10, IL-12, IL-13, IL-15, IL-17, IL-1beta, IL-1RA, IL-2, IL-2R, IL-4, IL-5, IL-6, IL-7, IL-8, IP-10/CXCL10, MCP-1/CCL2, MIG/CXCL9, MIP-1alpha/CCL3, MIP-1beta/CCL4, RANTES/CCL5, TNF-alpha, and VEGF decreased significantly after fish oil intake [61]. 

### 10.3. Multiple Sclerosis

Multiple sclerosis (MS) is characterized by an inflammatory neurodegenerative condition of the central nervous system which is typical of young and adult female subjects; in a minor percentage of cases it affects men. As in other diseases, in MS oxidative stress also is thought to be a key player along with chronic inflammation [69].

Oxidative stress is an important factor for the pathogenesis of this ailment. Its part in MS is connected to accelerated production of a few types of ROS, mainly through macrophages as the main factors responsible for demyelination and axonal injuries. Both the demyelination and the inflammation processes are connected to producing ROS [90].

A significant increase of total antioxidative status (TAS) level in plasma was noted in MS patients undergoing whole-body cryotherapy (WBCT) treatment. This indicates that WBCT might be a therapy which suppresses oxidative stress in MS patients. WBCT is a relatively new method of treatment, and is more and more often used in neurological ailments due to its analgesic behavior, muscle tone relief, and antidepressant effect, increasing the efficiency of kinesiotherapy. In MS pathophysiology, oxidative stress plays a major role. This is why seeking antioxidative therapies is key for treating MS patients. This study determined the level TAS in plasma and the activity of superoxide dismutase (SOD) and catalase (CAT) in erythrocytes of MS patients after 10 exposures of WBCT. Additionally, some of MS patients undergoing WBCT were supplemented with melatonin [62]. In subjects affected by MS, CoQ10 dietary intake (500 mg/day) was effective in decreasing oxidative stress and increasing antioxidant systems and demonstrated reduced lipid peroxidation, augmented E vitamin disposal, and balanced NO-related signaling. A direct result of these effects was neuroprotection [57]. In another study, CoQ10 supplementation was also found to improve fatigue and depression in patients with MS [58].

According to Sowa et al., ceruloplasmin was high in novel diagnosed MS subjects. In addition, ceruloplasmin level was related to the diverse immunomodulatory treatments administrated. Mitoxantrone and interferon beta 1b were linked to elevated ceruloplasmin levels. However, 12 weeks of melatonin intake ameliorated the protein levels in the control group. Ceruloplasmin was not connected to the phase of the disease. After treatment with interferon beta 1b and glatiramer acetate, ceruloplasmin diminished [63].

Administration of probiotics for 3 months in MS patients improved the expanded disability status scale (EDSS), parameters of mental health, inflammatory factors, markers of insulin resistance, HDL-, total-/HDL-cholesterol, and MDA levels [65]. For the same time period, other authors treated MS subjects with ω-3 fatty acid and vitamin D3 with positive outcomes on their disability score, inflammation and antioxidant capacity, and metabolic status. Other inflammatory parameters and oxidation markers were ameliorated. Among these were serum hs-CRP, plasma TAC, GSH, MDA, insulin metabolism, HDL-, and total/HDL-cholesterol [69].

### 10.4. Diabetes Mellitus

Diabetes mellitus is one of the most threatening diseases of the modern world due to its diffusion and short- and long-term consequences. It is characterized by a complete or partial lack of insulin secretion and/or insulin metabolism which results in chronic hyperglycemia. Ocular, renal, and cardiovascular pathologies are only some of the long-term effects primarily due to hyperglycemia. Research over the last few decades proposes chronic hyperglycemia as a principal causative agent of ROS production [75].

In particular, DM-1 is determined by gradual and irreversible immune-associated damage of insulin-producing pancreatic β cells. Hyperaggregability of platelets was intimately linked to lipid peroxidation products, like MDA in DM-1. Vitamin E (100 IU/day) was effective in reducing blood markers of hyperaggregability and lipid peroxidation [70].

Impaired endothelial vasodilator function (EVF) is related to low density lipoprotein (LDL) and vitamin E content (VEC) in patients with type 1 DM, highlighting the importance of oxidative stress in the disease. Vitamin E intake ameliorated vascular parameters in young DM-1 patients. This result was mostly connected to better LDL, VEC, and diminished oxidative vulnerability to LDL [91].

ROS and vessel damage are often linked in DM patients. A direct consequence is DNA damage in peripheral blood lymphocytes, as demonstrated by the comet assay. Vitamin E administration for 3 months improved the antioxidative response [71].

Wold et al. suggested the occurrence of augmented oxidative stress in diabetes due to the clear alterations in coenzymes Q9 and Q10 levels. IGF-1 supplementation abolished this effect, except for Q9 levels in the liver. The authors speculated that IGF-I could be useful in avoiding hyperglycemia-induced organ damage and oxidative stress in DM patients with comorbidities [27].

High-dose vitamin E administration (1200 mg/day) reduced markers of oxidative stress and improved antioxidant defenses but unfortunately had no effect on improving microalbuminuria (MA) [72].

Phytotherapeutic substances were also studied. Many of them were known to have hypoglycemic effects and antioxidant capacities. *Allium sativa*, *Ricinus communis*, *Securinega virosa*, and *Cassia auriculata* were administrated by ancient populations. Two months of supplementation with blueberry (*Vaccinium myrtillus*) concentrate augmented the antioxidant response in young type 1 diabetic patients. SOD and GPx augmented the antioxidant response as well. The phytotherapics had hypoglycemic results also, with a consequent need for insulin-treatment calibration [67].

Tsai et al. noticed that a diet including glutamin (Gln) did not modify plasmatic glucose. The inflammatory infiltration was lower and the GSH/GSSG ratio was augmented. In particular, leukocyte adhesion molecules, organ nitrotyrosine concentrations, and liver neutrophil infiltration were affected by Gln intake [79].

Gupta et al. suggested that vitamin E ameliorated oxidative stress in DM-1 subjects. They speculated that it improves antioxidant defenses. MDA levels were higher in DM1 patients. The supplementation decreased MDA levels and augmented GSH [73].

A study was conducted on young adult males with uncomplicated type 1 diabetes to verify the effects of supplementation with l-arginine (7 g/day) on endothelial function and on markers of oxidative stress. The authors examined L-arginine actions in terms of vascular and antioxidant protection. Oxidative stress/damage markers, carbonyls, and thiobarbituric acid reactive substances (TBARS) were increased in DM patients and L-arginine administration improved blood flow but did not influence the oxidative status [55].

Zinc supplementation was studied in an experimental animal model showing improved renal function in diabetic mice. It reduced the lipid peroxidation status in the kidney. As a consequence, the authors hypothesized that zinc supplementation could help moderate DM progression and effects. The diminution of Zn and Zn-dependent antioxidant enzymes could be one of the main causes of DM oxidative damages. Zinc was also reported to induce metallothionein (MT) gene expression, boosting antioxidant performance [84].

Shivanna et al. showed the effects of Streptozotocin-induced diabetes. Powdered stevia leaves and the extracted polyphenols were potent boosters of plasmatic glucose metabolism, with a reduction of ALT and AST in diabetic rats. Their administration also affected MDA reduction and supported antioxidant defenses [76].

The role of the thioredoxin interacting protein (TXNIP) was also evaluated. It was found to have a role in the pathogenesis of nonalcoholic fatty liver disease (NAFLD) in diabetes. The results obtained through in vivo and in vitro studies have shown that quercetin and allopurinol suppress the activation of inflammasome 3 containing the pyrine domain (NLRP3) and reduce the levels of interleukin-1 (IL-1β) by blocking overexpression of the hepatic protein interacts with the thioredoxin TXNIP and then modulates the expression of lipid metabolism genes in high glucose conditions. These data support the evidence that inhibition of hepatic TXNIP contributes to the alleviation of liver inflammation and lipid accumulation in type 1 diabetes. The effects on hepatic TXNIP caused by quercetin and allopurinol might have some implications for the avoidance of type 1 diabetes-associated non-alcoholic fatty liver disease (NAFLD) [77].

Regular intake of olive oil, has been associated with many health benefiting effects experienced by Mediterranean populations [92]. Administration of olive leaf powder has been shown to play a role in the suppression and production of proinflammatory mediators and NO-associated oxidative stress. Preventing hypoglycemia and augmenting enzyme antioxidants activities are examples of its effects. Olive leaf powders demonstrated diminishing kidney damage by reducing immune response, inflammation, and oxidative stress [81].

Probiotics proved their efficacy in DM treatment when Purano et al. tested Kefir, a probiotic fermented milk, on diabetic animals. The expression of inducible NO synthase (iNOS) was reduced in the kefir group, indicating improved glucose metabolism and a better antioxidant capacity [83].

Some authors evaluated the ability of CoQ10 to interfere with antimicrobial peptides and natural killer (NK) cells; in fact, these components are involved as causative agents of DM and its long-terms effects. These parameters were measured in diabetic patients after 3 months of CoQ10 administration. Plasmatic human cathelicidin antimicrobial peptide (CAMP) and human beta defensin 1 (hBD1), both antimicrobial peptides, were diminished in DM-1 subjects. CoQ10 did not affect them. The enzyme moderated the quantities of circulating hBD2 and stimulated modification of the subset distribution and activation markers in peripheral NK cells [59].

Isoflavones and alpha-galactooligosaccharides in soy exert antioxidant and anti-inflammatory actions. Fermented soy permeate (FSP) diminished oxidative stress and inflammation in diabetic animals. FSP integration regulated levels of carboxymethylisine (CML) and antioxidant enzyme activity and augmented Mn-SOD. Other pro-inflammatory mediators were also reduced after the test [78].

The role of lichens as a new bio-resource for natural antioxidants was also evaluated. In particular, the effects of the lichen species *Cetraria islandica* and *Pseudevernia furfuracae* were tested in diabetic conditions. The results revealed that these lichen species could be used safely within a certain dose range. Furthermore, *C. islandica* extracts showed important results compared to the *P. furfuracae* extract for antioxidant capacity. However, the protective effects of the *C. islandica* extract were insufficient against diabetes-induced pancreatic damage through the formation of oxidative stress. In conclusion, the use of *C. islandica* could serve as a prompt intervention in the reduction of the risk of type 1 diabetes [80].

The therapeutic potential of *Origanum vulgare* L. ssp. hirtum leaf extract (Greek oregano) was also evaluated. Rich in biophenols, methanolic oregano extract (MOE) for the treatment of T1DM was examined. The extract reduced the disease occurrence and conserved normal insulin release. Oregano was effective in reducing ROS and NOS. Once more, the antioxidant capacity occurred side-by-side with the anti-inflammatory action. Th17, Th2, and Treg were fundamental in this process. Secondarily, MOE protected B-cells from in vitro apoptosis by blocking caspase-3. Lastly, rosmarinic acid, a main component in MOE, only demonstrated incomplete protection from diabetes induction. Thus, MOE prevented mice from diabetes spread [82].

There is a positive association between an antioxidant diet supplemented with alpha-lipoic acid and a decrease in endothelial dysfunction in pediatric patients with T1DM. This was demonstrated after dividing 71 children into three groups: those on an antioxidant diet + alpha-lipoic acid, those on an antioxidant diet + placebo, and controls. A substantial decrease in bolus insulin was observed. It was therefore assumed that alpha-lipoic acid has an antioxidant influence in pediatric diabetes patients by decreasing insulin [54].

It is well known that diabetic patients could have cardiovascular damage as a consequence of endothelial dysfunction caused by hyperglycemia which in turn provokes oxidative damage, sustaining inflammation. Vitamins C and E might ameliorate endothelial function (EF), but some results were contrasting [74].

Hossein Nia et al. examined if clinoptilolite and nano-sized clinoptilolite decrease hyperglycemia and oxidative stress in animals affected by DM. They observed that nano-sized clinoptilolite had a hypoglycemic effect with no substantial influence on the markers of oxidative stress [75]. 

## 11. Antioxidants in Autoimmune Diseases

Reactive oxygen species (ROS) are commonly known as “oxidants”. They have the ability to act in their initial form or to react with other molecules, leading to a generation of other ROS. They can interact with several compounds such as lipids, proteins, and DNA. Augmented levels of ROS compromise normal life-processes in the human body, contributing to the pathogenesis of several diseases. An oxidant–antioxidant disequilibrium is detrimental for lipids, proteins, and DNA. Lipid peroxidation can irreversibly compromise cell membrane integrity, affecting its fluidity. This process also diminishes hydrophobicity of the cell membrane and augments its permeability. The normal consequence of this impaired structure is a fracture. On the other hand, oxidation of proteins also compromises their structure. The formation of additional bonds results in altered or destroyed enzyme functionality [3]. The hostile nature of ROS contributes to the promotion of inflammation, apoptosis, and necrosis by changing the DNA chain and by perturbating its stability. Two consequences of this are the proliferation of cells and fibrosis. The result of excessive ROS generation also includes the activation of transcription factors, such as NF-kB or AP-1 proteins and pro-inflammatory mediators [93].

Antioxidant mechanisms constituted by enzymes such as SOD, CAT, GPx, and MPO are not considered in this review. We focused on exogenous antioxidants able to transform ROS into less harmful molecules. Most of these compounds have been studied for several years and include the E and C vitamins, carotenoids, and flavonoids [7].

Vitamin E is an effective antioxidant that has 8 isoforms. It preserves against lipid peroxidation, converting more innocuous reactive radicals to tocopherol. Some research has demonstrated the presence of doubled a-tocopherol concentrations in non-asthmatics compared to asthmatic subjects. Vitamin C exerts its action in collaboration with vitamin E. It eliminates excessive radicals and restores vitamin E to its original form. Carotenoids include b-carotene, which is introduced in the diet (i.e., carrots, lettuce, fruit). Once in the body, it is transformed into retinol (fundamental for eye physiology). Retinol protects against singlet oxygen. Flavonoids are typical components of plants (such as green tea, leaves, and grapes).

### 11.1. Oxidative Stress, Damage, and Immune Recruitment

The role of oxidative stress in immune system diseases was individuated as an imbalance between over-production of reactive oxygen species (ROS) or nitrogen (RNS) generated by pro-inflammatory immune cells (such as macrophages, eosinophils and monocytes) and a reduced production of antioxidants with an alteration of defense systems like CAT, SOD, GSH [94]. The emblem of this imbalance is shown by Myeloperoxidase (MPO) behavior. MPO is a glycosylated heme-enzyme which is stored in neutrophils and macrophages azurophilic granules; these granules could exert a huge bactericidal action due to their content. In fact they contain hypochlorous acid generated from hydrogen peroxide and chloride ions [1]. Serum levels of MPO, when increased, are used as a diagnostic marker for some diseases because it could favor the inflammation typical of as asthma, atopic dermatitis, rheumatoid arthritis, and sometimes cancers [1,94].

### 11.2. Exogenous Antioxidants and Autoimmunity

Several molecules and products were tested in order to demonstrate or confirm their antioxidant nature in autoimmune diseases. Although the results are promising, several of these exogenous antioxidants are already widespread in clinical practice [95,96]. Some researchers conducted their experiments by using probiotics, based on Lactobacilli and Bifidobacteria. The modification of gut microbiota was demonstrated to be promising for several diseases interfering with the immune response. Recent data also reported how microbiota could influence epigenetics and consequently inflammatory status. Probiotic administration together with a permanent gut microflora change could constitute a future therapeutic strategy both for cancer and immune-related diseases [97,98]. However, the exact mechanism involved in their antioxidant ability still remains to be clarified. It is likely due both to a direct anti-ROS function and to an indirect anti-inflammatory aptitude for preventing cellular damage and ROS release. Flavonoids also were demonstrated being promising in blocking immune system self-damage in autoimmune diseases. They act both by reducing inflammation and by regulating immune response playing at different levels. Their ability to block NF-kB was reported, therefore inhibiting the release and production of detrimental pro-inflammatory mediators. The most obvious consequence is the avoidance of further damages induced by oxidative stressors like NO and ROS [24]. 

More specifically, the autoimmune diseases analyzed shared some pathogenetic mechanisms and differed in others. The dietary supplementations reduced pro-inflammatory cytokines in RA and SLE [99,100]. 

### 11.3. ROS, the Immune System, and Inflammation

**Figure 1:**
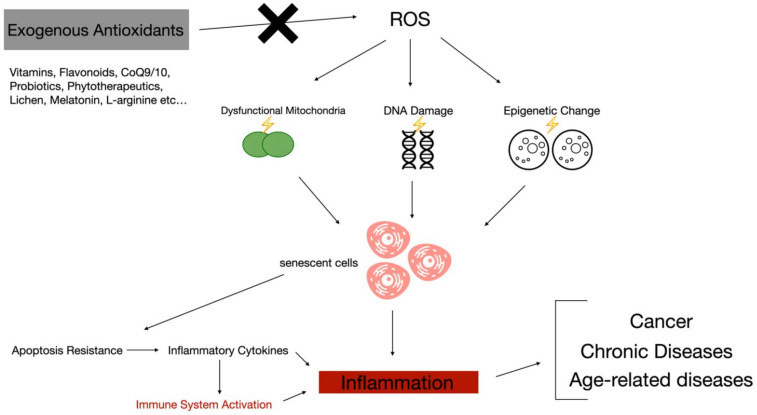
Some of the most studied exogenous antioxidants blocking reactive oxygen species (ROS) cascade in triggering and sustaining chronic inflammation and immune-related diseases.

Ever-increasing evidence of intimate connections between oxidative stress and chronic diseases has been reported. It was also demonstrated that oxidative stress plays an important role in cellular aging and as promoter of cancer [101]. Oxidative stress also favors tissue relapse of inflammation mediators through upregulation of their genes Immune system cells in turn, once activated, release pro-inflammatory cytokines, creating a vicious cycle [102]. Due to its inflammatory capability, oxidative stress could be of main importance in the pathogenesis of immune-related diseases. Often in autoimmune diseases there is a continuous recruitment and activation of inflammatory cells. ROS are over-generated often as a consequence of exposure to the external and internal causal agents that favor the onset of these pathologies. Free radicals, like superoxide anions and hydrogen peroxide, interfere with leukocytes (and the others cells) influencing immune function. Moreover, there is an expansion of ROS presence that occurs after the intervention and the activation of chronic inflammation, leading to a vicious cycle. This perpetuation of the stimuli and of the immune system activation in a sort of loop does seems to happen in acute pathologies [103]. There is a correlation between mitochondrial dysfunction and increased oxidative stress and the onset of autoimmune diseases. Mitochondria have a main role in many phases of the immune response; some of these fundamental processes are dendritic cell differentiation, antigen presentation, T-cell stimulation, and B-cell hyper-proliferation and activation [104]. This cascade leads to the release of pro-inflammatory cytokines (Figure 1). Antioxidants could have beneficial effects on autoimmune-related inflammation in humans; in this scenario, various nutritional compounds are capable of influencing the metabolic balance by equilibrating mitochondrial activity and ROS generation. The bidirectional cause–effect theory of the connection between mitochondrial metabolic stress and pathogenesis of autoimmune inflammation could in part explain the results that emerged from the literature.

### 11.4. The Allergic Lesson: Antioxidant Administration as Potential Therapeutic Approach

As mentioned above, antioxidants are enzymatic and non-enzymatic mechanisms able to counteract the damaging effect of oxidative stress. Internal constitutive antioxidants are constituted by enzymes which counterbalance ROS effects. The aim of these mechanisms is the change of radicals into less harmful molecules [105]. There are also non enzymatic and exogenous antioxidants which counterbalance oxidative stress. These compounds intake happen thanks to diet. Some examples were reported in this extensive review [106].

Several papers demonstrated antioxidant properties in vitro; however, their capabilities in vivo remain still uncertain. FeNO and other oxidative stress biomarkers in fact, reported a weak association between asthma and vitamin A and E intake benefits [107]. The role acted by dietary supplementation in the severity of asthma is still in progress. In neonatal studies, levels of selenium in maternal and cord blood predicted a minor incidence of wheeze [108]. The vantage of selenium presence in adults is still controversial. Animals had a worse outcome both with low and high selenium levels suggesting a complex role of selenium on asthma. Zinc and copper gave almost the same uncertain results [109].

It was shown Paeonia and Schisandra therapeutic effect and antioxidant activity in asthmatic disease. This extract seemed to influence the level of eosinophils, cytokines, NF-kB with an antioxidant trend. Saponins, lignans, flavonoids, and phenols are the most imputed antioxidant substances contained in the plant [110].

Nowadays, many dietary supplements are available for modifying oxidative stress, such as vitamins and minerals. Theoretically promising, dietary antioxidant drugs showed no significant benefits also in other allergic diseases. However, most studies have a high risk of bias due to the diversity of samples and diseases. However, most antioxidant supplements have no side effects. These dietary supplements are an economic burden for the patients in a long-term intake. More large scale and better-designed researches are necessary in order to fully consider the efficacy in allergic diseases [111,112,113].

## 12. Conclusions

Oxidative stress is a detrimental process that is physiologically present in human beings. This process is counterbalanced by several endogenous systems. Sometimes these protective systems are not enough and exogenous antioxidants are needed to support the innate ones. ROS cooperate in cellular damage typical of immune-related diseases and sustain additional pathological events once released after pro-inflammatory tissue disruption. This self-sustaining loop constitute one of the main checkpoints of autoimmune diseases and chronic inflammation. The administration of exogenous substances through food derivates or dietary supplements following scientific standardization was demonstrated to be effective. Further bias-free and extended studies should be conducted that include ever-increasing oxidative stress biomarkers. Antioxidant supplementation may delay the onset of some immune-related diseases and ameliorate the course of autoimmune diseases.

## Figures and Tables

**Table 1:** Clinical studies supporting the use of the exogenous antioxidant compounds (considered), in autoimmune diseases.

Exogenous Antioxidant (Compound)	Study Design	Study Protocol	Proncipal Endpoint	Observed Effect (Outcome)	Reference
Alpha-lipoic acid	Randomized, double-blind, placebo-controlled clinical trial	Patients affected by Rheumathoid arthritis (RA) (*n* = 70) (age range 20–50 years) were randomly assigned 1:1 to receive 2 capsules a day, every 12 h, 30 min prior to breakfast and dinner either Alpha lipoic acid (ALA) (1200 mg/day) or placebo (maltodestrin 600 mg) for 8 weeks.	Inflammatory biomarkers [serum high-sensitivity C-reactive protein (hs-CRP), tumor necrosis factor-alpha (TNF-α), interleukin-6 (IL-6), and serum matrix metalloproteinase-3 (MMP-3)]. Moreover, 3-day dietary records, the International Physical Activity Questionnaire (IPAQ), and the Spielberger State-Trait anxiety inventory form Y (STAI-Y) were evaluated before and after the intervention.	No statistically significant differences were observed in serum levels of hs-CRP, TNF-α, IL-6, and MMP-3 within and between the ALA and placebo groups (*p* > 0.05). There were no statistically significant differences in dietary intakes, physical activity, and anxiety levels between groups at baseline and during the study period (*p* > 0.05).	Mirtaheri et al., 2015 [53]
	Prospective, randomized, double-blind, controlled trial	Seventy-one children and adolescents (mean age 17 ± 3.9 yrs), with type 1 diabetes, were randomized into 3 arms: 10.000 oxygen radical absorbance capacity units (ORAC) antioxidant diet plus alpha-lipoic acid (400 mg) (*n* = 25, group 1), 10.000 ORAC antioxidant diet plus placebo (*n* = 27, group 2), and controls (*n* = 19, group 3), with no changes in dietary habits. Treatment was administered twice a day at least 1 h before lunch and dinner, for 6 months.	Improvement in endothelial dysfunction measured as mean of reactive hyperemia index (RHI)	At 6 months, RHI score was significantly improved only in the group of patients treated with antioxidant diet plus ALA ( *p* = 0.045), reaching a normal RHI value. The group of patients treated with antioxidant diet plus placebo reported a better RHI score at 6 months versus baseline as well, but it did not reach the significance. No differences in RHI score were showed in the control group at 6 months versus baseline.	Scaramuzza et al., 2015 [54]
Arginine	Randomized placebo controlled trial	Ten young adult male subjects with uncomplicated type 1 diabetes and twenty controls were enrolled (range age 18–30 years). Patients were supplemented with L-arginine (7 g/day during) or placebo (amide-based formulation) for 7 days.	Over lower limb blood flow, oxidative stress marker (TBARS, Carbonyls), anti-oxidant parameters (uric acid and TRAP) and total tNOx in rest conditions and after a single bout of submaximal exercise were evaluated.	L-arginine supplementation (7 g/day) caused a statistically significant improve of endothelial function (i.e., blood flow) in basal conditions in young adult subjects with non-complicated type 1 diabetes (*p* < 0.05).	Fayh et al., 2013 [55]
Coenzyme Q10	double-blind, randomized controlled clinical trial	44 patients with rheumatoid arthritis were recruited (age range 18–65 years). Patients received daily a 100 mg capsule of CoQ10) (*n* = 27) or placebo (wheat starch) (*n* = 27) for 2 months in addition to their conventional medications.	Measure malondialdehyde (MDA), total antioxidant capacity (TAC), interleukin (IL)-6 and tumor necrosis factor alpha (TNF-α).	Serum MDA significantly decreased in supplemented group (mean difference = -1.47 nmol/mL; 95% confidence interval (CI), −2.52 to −0.43; *p* = 0.008). CoQ10 also suppressed overexpression of TNF-α (difference in median was +1.1 in placebo vs. +0.03 in CoQ10 group; *p* = 0.033). There was no significant difference in TAC and IL-6 levels between groups.	Abdollahzaet al., 2015 [56]
	Randomized, double-blind, placebo-controlled clinical study.	48 patients with relapsing-remitting Multiple sclerosis (MS). Subjects were randomly assigned to a placebo group (mean age 30.9 ± 7.7 years) (*n* = 24) or coenzyme Q10 (CoQ10) supplemented group (mean age 33.1 ± 7.6 years) (*n* = 24) (500 mg/day). The intervention was administered for 12 weeks.	Measure inflammatory (tumor necrosis factor-α (TNF-α), interleukin (IL)-6, and matrix metalloproteinase (MMP)-9) and anti-inflammatory (IL-4 and TGF-β) markers.	Supplementation of CoQ10 caused a statistically siognificant decrease of TNF-α levels (*p* = 0.003). CoQ10 group showed significantly lower IL-6 levels (*p* = 0.037), compared to the placebo group. CoQ10 supplementation also resulted in decreased serum levels of MMP-9 as compared to the placebo group (*p* = 0.011). IL-4 and TGF-β levels were not modified by CoQ10 supplementation.	Sanoobar et al., 2015 [57]
	Randomized, double-blinded, placebo-controlled trial	48 patients were randomly allocated to two groups: CoQ10 group (*n* = 24; mean age 33.1 ± 7.6 years) and control (placebo) group (*n* = 24; 30.9 ± 7.7 years). The patients in CoQ10 group were treated with CoQ10 at doses of 500 mg per day for 3 months, whereas controls received placebo capsules (with the same shape and color of CoQ10 capsules). to determine the effect of CoQ10 supplement (500 mg/day) vs. placebo for 12 weeks.	Fatigue symptoms were quantified by means of fatigue severity scale (FSS) and the Beck depression inventory (BDI) was used to assess depressive symptoms.	A significant decrease of FSS was observed in CoQ10 group during the intervention (*p* = 0.001) and significant increase of FSS change was observed within placebo group (*p* = 0.001). Repeated measure analysis of variance showed a significant time-by-treatment interaction for FSS (*p* < 0.001) and BDI (*p* < 0.001), indicating significant decrease of FSS and BDI in CoQ10 group compared to placebo group.	Sanoobar et al., 2015 [58]
	Controlled clinical trial	A total of 58 patients with diabetes (*n* = 27, T1DM and *n* = 31, T2DM), and 19 healthy controls were recruited. (Mean age of the groups: T1DM, 53.2 ± 15.5 years; T2DM, 58.7 ±8 years; controls, 50.3 ± 14.8 years) A subset of 23 patients with diabetes received CoQ10 orally for 12 weeks (100 mg twice daily).	AMPs and NK cells in diabetes patients and the influence of CoQ10 on these immune components in the diabetic patients have been investigated.	Supplementation with CoQ10 improved NK cell activity and reduced hBD2 expression in T1DM patients. This suggests that the anti-oxidant CoQ10 also targets the immune system and especially improves T1DM-induced disorders.	Brauner et al., 2014 [59]
Fish oil/primrose oil	Prospective, randomized controlled trial	60 postmenopausal, female patients (mean age 63.1 ± 9.6 years) with rheumathoid arthritis (RA) were recruited and divided into three groups: group I control group (*n* = 20) only their strikethrough rheumatologic therapy; group II (*n* = 20) taking, daily after meals, five gel capsules of Omega-3 Cardio (containing 1000 mg of concentrated fish oil with 300 mg of docosahexaenoic acid (DHA), 200 mg EPA, 100 mg of other omega-3 fatty acids) in a period of 3 months with a regular rheumatologic therapy, group III (*n* = 20) taking, daily after meals, two gel capsules of Omega-3 Cardio and two gel capsules of evening primrose oil (containing 1300 mg of evening primrose oil with 949 mg of linoleic acid and 117 mg of gamma linolenic acid (GLA)) in a period of 3 months with a regular rheumatologic therapy.	The following oxidative stress markers have been evaluated: index of lipid peroxidation (thiobarbituric acid-reactive substances (TBARS)), hydrogen peroxide (H2O2), superoxide anion radical (O2 (-)), nitric oxide (NO), superoxide dismutase activity (SOD), catalase activity (CAT), and glutathione levels (GSH) in erythrocytes	No statistically significant changes for any of the oxidative stress parameters have been detected in group I. In group II, levels of TBARS, NO2 (-), and GSH were increased, while levels of H2O2 decreased. Increased values of TBARS, NO2 (-), and SOD were found in group III.	Vasiljevic et al., 2016 [60]
	A 6 month single center, randomized, single-blind (patient unaware of treatment group), placebo-controlled, parallelgroup pilot study.	50 patients affected by Systemic Lupus Erithematosus (SLE) (age range 18–64 years) recruited in outpatient clinics were randomized 1:1 to fish oil supplementation or olive oil placebo. (6 capsules/day equaling 2.25 g EPA and 2.25 g DHA) or placebo (6 capsules/day purified [refined, not extra-virgin] olive oil Metagenics) in addition to their background therapies, for 6 months.	RAND Short Form-36 (RAND SF-36), Fatigue Severity Scale (FSS), SLE Disease Activity Index (SLEDAI), and Physician Global Assessment (PGA) were evaluated.	PGA swas ignificantly improved in the fish oil group compared with the placebo group (*p* = 0.015). The RAND SF-36 Energy/fatigue and Emotional well-being scores demonstrated improvement trends (*p* = 0.092 and 0.070). No clear difference was seen in FSS and SLEDAI (*p* = 0.350 and *p* = 0.417). Erythrocyte sedimentation rate and serum IL-12 were reduced (*p* = 0.008 and *p* = 0.058) while serum IL-13 was increased by fish oil supplementation (*p* = 0.033).	Arriens et al., 2015 [61]
Melatonin		Patients affected by Multiple sclerosis (MS) (*n* = 28) before and after 10 exposures of *whole body cryotherapy* (WBCT; -120 degrees C/3 min/day. 16 MS patients during 10 exposures of WBCT additionally were supplemented by 10 mg of melatonin.	Total antioxidative status (TAS) in plasma and activity of superoxide dismutase (SOD) and catalase (CAT) in erythrocytes of MS patients	TAS level in plasma as well as supplemented with melatonin and non-supplemented MS patients was observed after 10 exposures of WBCT. Melatonin statistically significant increased activity of SOD and CAT in erythrocytes of MS patients treated with WBCT.	Miller et al., 2011 [62]
	Prospective study	102 patients affected by Multiple sclerosis (MS) and 15 healthy controls (mean age 36.45 ± 8.16 years) were enrolled. Patients were divided into groups according to different immunomodifying therapies: A (interferons beta 1a, mean age 41.22 ± 7.1); B (interferons beta 1b, mean age 39.48 ± 9.42), G (glatiramer acetate, mean age 38.95 ± 10.76); Mx (mitoxantrone, mean age 55.74 ± 6.21) and P (immunomodifying pre-treatment, mean age 37.33 ± 9.23).; R (relapse gropup, mean age 41.90 ± 7.13). MS patients were supplemented with melatonin (5 mg daily) for 3 months.	Serum ceruloplasmin concentrations, Expanded disability status scale score (EDSS), brain magnetic resonance imaging, serum C-reactive protein level, and white blood cell count were examined.	Significantly increased levels of ceruloplasmin in MS patients was detected (*p* = 0.0048). No differences in ceruloplasmin concentrations between the relapse group and controls were observed. In A and G groups, ceruloplasmin levels before and after melatonin were similar to levels in controls. In group B, ceruloplasmin concentration was significantly higher vs. control and relapse groups. After 3 months of melatonin administration in group B patients, the levels of ceruloplasmin decreased significantly (*p* = 0.012). No significant changes were observed between controls, pretreated group, and relapse group, and after melatonin treatment in the group G. after melatonin administration in the Mx patient group, was observed a ceruloplasmin levels decrease, but not statistically significant.	Adamczyk-Sowa et al., 2016 [63]
Probiotics	A Randomized Double-Blind Clinical Trial	46 patients with Rheumatoid arthritis (RA) were assigned to one of two groups; patients in the probiotic group (41.14 ± 12.65) received a daily capsule containing 10^8^ colony forming units (CFUs) of *Lactobacillus casei* 01 (L. casei 01), and placebo group (44.29 ± 9.77) took identical capsules containing maltodextri for 8 weeks.	Anxiety, physical activity levels, and dietary intakes were assessed. Anthropometric parameters, serum malondialdehyde (MDA), total antioxidant capacity (TAC), erythrocyte superoxide dismutase (SOD), glutathione peroxidase (GPx), and catalase (CAT) activities were measured.	No significant difference between the two groups for anthropometric parameters, physical activity, anxiety levels, or dietary intakes, throughout the course of the study were detected. No significant within- and between-group differences were observed for MDA, TAC, or CAT. SOD activity decreased only in the probiotic group and GPx activity decreased in both study groups (*p* < 0.05); no significant between-group difference was found for these enzymes activities at the end of the study (*p* > 0.05).	Vaghef-Mehrabany et al., 2016 [64]
Probiotics	Randomized double-blind placebo-controlled clinical trial	60 patients affected by Multiple sclerosis (MS). Participants were randomly allocated into two groups to receive either a probiotic capsule (*n* = 30; mean age 33.8 ± 8.9) or placebo containing starch (*n* = 30; mean age 34.4 ± 9.2) for 12 weeks. The probiotic was containing *Lactobacillus acidophilus, Lactobacillus casei, Bifidobacterium bifidum* and *Lactobacillus fermentum* (each 2 × 10^9^ CFU/g).	Expanded disability status scale (EDSS) scoring, parameters of mental health and metabolic indicators were recorded at the baseline and 12 weeks after the intervention.	Probiotic intake improved EDSS (*p* = 0.001), beck depression inventory (*p* < 0.001), general health questionnaire (*p* < 0.001), depression anxiety and stress scale (*p* = 0.001). Changes in high-sensitivity C-reactive protein (*p* = 0.01), plasma nitric oxide metabolites (*p* = 0.002) and malondialdehyde (MDA) (*p* = 0.04) were also significantly different compared to placebo group. Moreover probiotic capsule significantly decreased serum insulin (*p* < 0.001), homeostasis model of assessment-estimated insulin resistance (*p* = 0.001), Beta cell function (*p* < 0.001), Total-/HDL-cholesterol (*p* = 0.02). Quantitative insulin sensitivity check inde, (*p* < 0.001) and HDL-cholesterol levels (*p* = 0.02), were also significantly increased.	Kouchaki et al., 2017 [65]
Sesamin	randomized, double-blind, placebo-controlled clinical trial	44 patients (age range: 18–55 years) with Rheumatoid arthritis were randomly divided into 2 groups (intervention and control). Patients consumed 200 mg/day sesamin supplement and placebo in the intervention and control groups, respectively, for 6 weeks.	Anthropometric indices and blood pressure were assessed. Serum concentrations of lipid profile, malondialdehyde (MDA), and total antioxidant capacity (TAC) were also determined.	Sesamin supplementation significantly decreased serum levels of MDA (*p* = 0.018) and increased TAC and high-density lipoprotein cholesterol levels in patients with RA (*p* = 0.020 and *p* = 0.007, respectively). In the sesamin group, the mean of weight, body mass index, waist-to-hip ratio, body fat, systolic blood pressure, and the concentration of triglycerides, total cholesterol, and low-density lipoprotein cholesterol were also significantly decreased compared to baseline values (*p* < 0.05). However, the difference between the 2 groups was not statistically significant in this regard (*p* > 0.05).	Helli et al., 2016 [66]
*Vaccinium myrtillus*	Placebo controlled trial	30 children (age range 8 to 17 years) suffering from type 1 diabetes. In the first two months patients were given a placebo. In the next two months the dietary supplement was administered 3 × 1 comprimates/day, before meals.	Glycemia values (measured in the morning, before the first dose of insulin), glycated hemoglobin (HbA1C), C peptide level and changes in antioxidant enzyme activity after two months of treatment with the dietary supplements were evaluated.	Two months of supplementation with blueberry (*Vaccinium myrtillus*) concentrate augmented the antioxidant response in young type 1 diabetic patients. SOD and GPx augmented the antioxidant response as well. The phytotherapics had hypoglycemic results too, with a consequent need for insulin-treatment calibration.	Nemes-Nagy et al., 2008 [67]
Vitamin C	Randomized, double blind, placebo controlled pilot study	39 Patients affected by Systemic Lupus Erithematosus (SLE) (mean age was 46 ± 9 years) were randomized to receive either vitamins (*n* = 20; mean age 44 ± 6 years) 500 mg vitamin C and 800 IU vitamin E daily) or placebo (*n* = 19; mean age 48 ± 11 years) for 12 weeks.	Malondialdehyde (MDA) and allantoin were evaluated. Erythrocyte superoxide dismutase and glutathione peroxidase, plasma total antioxidant power (as FRAP value), and ascorbic acid and vitamin E concentrations were also measured. Endothelial function was assessed by flow-mediated dilatation (FMD) of the brachial artery and plasma concentration of von Willebrand factor (vWF) and plasminogen activator inhibitor type 1 (PAI-1).	Plasma ascorbic acid and α-tocopherol concentrations were significantly (*p* < 0.05) increased only in the vitamin-treated group, associated with a significant decrease (*p* < 0.05) in plasma MDA. Other oxidative stress markers and antioxidant levels remained unchanged in both groups. FMD and vWF and PAI-1 levels remained unchanged in both groups.	Tam et al., 2005 [68]
Vitamin D	randomized, double blinded, placebo-controlled clinical trial	53 Patients affected by Multiple sclerosis (MS), were recruited and divided into 2 groups to receive either 2 ω-3 fatty acid capsules daily (containing 500 mg DHA and 106 mg EPA) plus vitamin D3 as cholecalciferol supplements (50,000 IU/biweekly) (*n* = 26; mean age 33.3 ± 6.5 years) or sunflower oil capsules (placebo, *n* = 27; mean age 35.2 ± 9.2 years) for 12 weeks.	The primary outcomes of the study included Expanded disability status scale (EDSS) score and inflammatory markers. Biomarkers of oxidative stress and metabolic profiles were the secondary outcomes of the study.	Coadministration of ω-3 fatty acid and vitamin D3, for 12 wk, significantly decreased EDSS score (*p* = 0.01) in patients with MS. Serum hs-CRP (*p* < 0.001), plasma TAC (*p* = 0.02), GSH (*p* = 0.007), and MDA (*p* < 0.001) improved significantly in the supplemented group, compared with the placebo group. A significant reduction in serum insulin (*p* = 0.008), HOMA-IR (*p* = 0.01), and total/HDL-cholesterol (*p* = 0.04) was also reported. A significant increase in QUICKI (*p* = 0.008) and serum HDL-cholesterol concentrations (*p* = 0.009) compared with the placebo was also reported.	Kouchaki et al., 2018 [69]
Vitamin E	Double-blind, placebo controlled clinical trial	Diabetic patients (*n* = 29; mean age 12.7 ± 0.8 years) and healthy control (*n* = 21; mean age 10.9 ± 0.9) were recruited for the study. Diabetic patients were supplemented with DL-a-tocopherol (vitamin E) capsule (orally, 100 IU/day) or placebo for 3 months. Alternate diabetic patients were assigned to vitamin E or placebo during regular visits to the clinic.	Platelet aggregability was assessed by assay of the blood TxB2. Plasma vitamin E and MDA (malondialdehyd) levels were assessed.	Vitamin E supplementation lowered MDA levels by 30% (*p* < 0.04), TxB2 levels by 51% (*p* < 0.03), and triglyceride levels by 22% (*p* < 0.04) in diabetic patients.	Jain et al., 1998 [70]
	Placebo controlled trial	60 diabetic patients were assigned to receive Vitamin E (a-tocopherol) 900 mg/daily (*n* = 32, mean age 45.7 ± 17) or placebo (*n* = 28; mean age 44.17 ± 11.5) for 12 weeks.	DNA damage (based on extend of DNA migration)	Supplementation of Vitamin E significantly decrease in DNA migration in all 32 treated patients compared with their mean cells with migration before treatment (*p* < 0.05).	Sardas et al., 2001 [71]
	Randomized, placebo-controlled, double-blind cross-over trial	Ten young adults (mean age 18.87 +/− 2.91 years) with T1DM and persistent microalbuminuria (MA) were recruited. The vitamin E was encapsulated in oval and soft elastic gelatine (each capsule contained 300 mg D-α-tocopherol acetatedissolved in 100 mg of edible vegetable oil. Placebo capsules contained 500 mg of soybean oil dissolved in the same 100 mg of vehicle. Treatment was administerd for 12 months.	Evaluation of the effects of high-dose vitamin E supplementation on reducing both MA and oxidative stress in patients with type 1 diabetes mellitus and persistent MA. Determination of albumin excretion rate (AER) and HbA1c and evaluation of the oxidant/antioxidant status were performed.	No differences in terms of oxidant and antioxidant status were found between the two groups. This was associated with no significantly different urinary VEGF and TGF-beta levels. After 6 months, no significant differences in AER were observed between the two groups (*p* = 0.59). However, plasma and LDL-vitamin E content were significantly higher in the vitamin E group compared to the placebo group (*p* = 0.0001 and *p* = 0.004, respectively). This was associated with a significantly longer lag phase (*p* = 0.002) and lower MDA (*p* = 0.049). However, no statistically significant differences were detected in terms of VEGF and TGF-beta urinary levels.	Giannini et al., 2007 [72]
	Controlled clinical trial	40 children (20 Type 1 Diabetes Mellitus patients and 20 healthy controls) were enrolled. Patients were supplemented with 600 mg/daily vitamin E for three months.	Oxidative stress parameters malondialdehyde (MDA), antioxidants, reduced glutathione (GSH), vitamin E and metabolic parameters were evaluated before and after the period of supplememtation.	Vitamin E supplementation caused a significant decrease in MDA levels and significant increase in GSH (*p* < 0.05) and vitamin E (*p* < 0.05) levels in diabetic patients No significant changes were observed in metabolic parameters in Type 1 Diabetes Mellitus patients after vitamin E supplementation (*p* > 0.05).	Gupta et al., 2011 [73]
	open-label	Nine children and adolescents (age range 8–15 years; mean age Mean age was 12.9 ± 0.9 yr) with T1DM were included in this study. Open-label antioxidant supplementation was given for six weeks.The doses were chosen to be equivalent to a dose of 1 g of vitamin C and 400 IU of vitamin E per day in a 75 kg adult. (< 30 kg; Vit C 250mg; Vit E100 IU); (30–60 kg; Vit C 500mg; Vit E 200 IU); (> 60 kg; Vit C 750mg; Vit E 300 IU).	Endothelial function and plasma measurements of biochemical endothelial risk antioxidant capacity, inflammatory markers (CRP and IL6) were evaluated. Effect on endothelial colony forming cells (ECFCs: CD34+ CD133+ CD45−) was also considered.	No differences were seen in the forearm vascular resistance response to occlusion between before and after combined vitamin C and E supplementation. No differences were observed in hsCRP, total plasma antioxidant capacity (TAOC), adiponectin, or endothelial progenitor cells before or after vitamin C and E supplementation.	Cazeau et al., 2016 [74]

**Table 2:** Pre-clinical study carried out with selected compounds in experimental models of autoimmune diseases.

Exogenous Compound	Route of Administration	Species	Experimental Model/Dosage	Observed Effects	Reference
Clinoptilolite	Oral administration	Male Wistar rats	Thirty-six rats were randomly allocated to 2 groups. 1 group was randomly chosen to be a diabetic group and injected with streptozotocin (60 mg/kg body weight in 0.1 mol/L sodium citrate buffer). Three days after diabetes induction, each group was randomly divided into 3 subgroups of 6 animals each ([1] control, [2] 1% cliniptilolite (CLN)/food, [3] 1% nano sized CLN (NCLN)/food). Animals were fed a diet containing 1% powdered CLN or NCLN and a normal diet for 4 consecutive weeks.	Blood glucose and malondialdehyde were significantly elevated. No statistically significant changes in superoxide dismutase, glutathione peroxidase or total antioxidant capacity in diabetic rats have been shown. In diabetic rats treated with NCLN, blood glucose decreased to near normal levels (12.4 vs. 27.5 mmol/L). No significant changes were found in the other groups. None of the oxidative stress indices showed significant changes in either the treated or untreated rats.	Hossein et al., 2018 [75]
Flavonoids	Intragastric gavage	Male Wistar rats	Eighty rats were randomly divided into 8 groups of 10 rats each. Groups 1 and 2 were fed with the control diet; groups 3 and 4 with 4.0% *Stevia rebaudiana* leaves powder incorporated diet (4.0 g leaf powder in 96 g dry diet); groups 5 and 6 with equivalent amount of polyphenols extract (through force feeding); groups 7 and 8 with equivalent amount of fiber extracted from 4 g of *Stevia rebaudiana* leaves powder respectively for 5 weeks.	Results showed a reduction of blood glucose, ALT and AST, and increment of insulin level in the stevia whole leaves powder and extracted polyphenols fed rats compared to control diabetic group. Its feeding also reduced the MDA concentration in liver and improved its antioxidant status through antioxidant enzymes. Glucose tolerance and insulin sensitivity were improved by their feeding. Moreover in the *Stevia rebaudiana* leaves and extracted polyphenol fed groups an improvement of streptozotocin kidney damage has been shown.	Shivanna et al., 2013 [76]
	Intragastric gavage	Male Sprague-Dawley rats Rat BRL-3A and human HepG2 cells.	Quercetin and allopurinol were suspended in water and administered via intragastric gavage (1 mL·100 g^−1^ body weight). The treatment was started on day 4 after STZ injection by gavage once daily for 7 weeks. In vitro studies were also performed. Cells were cultured in medium containing low-glucose DMEM (5.5 mM) and 10% FBS. Cells were maintained at low glucose (5.5 mM, as control) or high glucose (30 mM) in the presence or absence of 10–20 μM quercetin, 100 μM allopurinol or 20 μM of the caspase-1-specific inhibitor Ac-YVAD-CMK (Ac-Tyr-Val-AlaAsp-chloromethylketone) for 24, 48 or 72 h.	Quercetin and allopurinol significantly inhibited the TXNIP overexpression, activation of NOD-like receptor 3 (NLRP3) inflammasome, down-regulation of PPARα and up-regulation of sterol regulatory element binding protein-1c (SREBP-1c), SREBP-2, fatty acid synthase and liver X receptor α, as well as elevation of ROS and IL-1β in diabetic rat liver. These effects were confirmed in hepatocytes in vitro and it was further shown that TXNIP down-regulation contributed to the suppression of NLRP3 inflammasome activation, inflammation and changes in PPARα and SREBPs.	Wang et al., 2013 [77]
	Intragastric gavage	Male Wistar rats	Diabetic induced animals received daily, water (placebo groups) or diluted fermented soy permeate (FSP 0.1 g/day; FSP-supplemented group) for 3 weeks. FSP is a soluble powder containing 29% alpha-GOSs (alpha-linked galactooligosaccarides) and 0.5% soy isoflavones (daidzein and genistein).	FSP supplementation in diabetic animals normalized the CML and antioxidant enzymatic activity levels and tended to increase Mn-SOD expression. IL-1b, IL-6 and uric acid, were markedly decreased by FSP supplementation.	Malarde et al., 2015 [78]
Glutamin	Normal feeding	Male Wistar rats	Diabetic control group (DM) was fed a common semipurified diet, and another diabetic group received a diet in which part of the casein was replaced by Glutamine (Gln; DM-Gln), which provided 25% of the total amino acid nitrogen for 8 wk. Diabetes was induced by an intraperitoneal injection of nicotinamide (NA) 150 mg/kg followed by STZ.	Gene expressions of transforming growth factor-b1 and interleukin-17A did not differ in blood mononuclear cells among the three groups. Expressions of interleukin-6, interleukin-23, monocyte chemotactic protein-1, and the receptor of the advanced glycated endproducts gene were higher in blood mononuclear cells and the ratio of reduced to oxidized glutathione was lower in erythrocytes in the DM group than in the normal control group. mRNA expressions of these genes were lower, whereas the ratio of reduced to oxidized glutathione was higher in the DM-Gln group than in the DM group.	Tsai et al., 2012 [79]
*Lichen Species*	Intraperitoneal administration	Male Spraque Dawley rats	Diabetic rats were treated with aqueous lichen extracts (250 and 500 mg/kg/day) for 2 weeks. The extracts were obtained from *C. islandica* and *P. furfuracea* lichen species	Blood glucose and insulin levels were not affected by Lichen extracts supplementation. *P. furfuracea* extract increased the level of antioxidant enzymes at both dosages (250 and 500 mg/kg) but the best result was observed at the doses of *C. islandica*. However, the increases of SOD and CAT activities were not significant in induced diabetic groups and oxidative stress did not return to the control levels, moreover the protectivity of *C. islandica* did not improve diabetes-induced pancreatic damage.	Çolak et al., 2016 [80]
Olive leaf powder		Male mice	80 Mice were randomly assigned to 5 groups (C) control; (D) diabetes; V ery low dose of olive leaf powder (VLOL, diabetes + 0.3% olive leaf powder); low dose of olive leaf powder (LOL, diabetes + 0.6% olive leaf powder); and high dose of olive leaf powder (HOL, diabetes + 3% olive leaf powder). Animals received the treatment for 4 weeks.	SOD, CAT and GPx were increased in the VLOL and LOL groups. Nitric oxide levels decreased in the VLOL and LOL groups, compared with the D group. mRNA expression levels of iNOS were significantly decreased in the VLOL and HOL groups, and IFN-γ levels were significantly decreased in the liver of the VLOL, LOL, and HOL groups compared with the levels of D group. IL-17 levels were significantly decreased in the VLOL and HOL groups. Th1 and Th17 cytokine levels were increased in the D group but decreased in all the experimental groups. Th2 cytokine levels were increased in all olive leaf–supplemented groups compared with those in the D group.	Park et al., 2013 [81]
*Origanum vulgareOriganum vulgare L. ssp. hirtum* (Greek oregano) leaf extract rich in biophenols.	Intraperitoneally injection	C57BL/6 mice	Methanolic estractc (MOE) or acqueous extrac (AOE) were administered for ten consecutive days by intraperitoneal injections at a dose of 5 mg/kg per d, starting from the day of diabetes induction (prophylactic’ regimen) or starting 1 d after the last streptozotocin injection (early therapeutic). Rosmarinic acid was administered intraperitoneally for ten consecutive days (25 mg/kg per d), starting from the day of diabetes induction.	MOE reduced diabetes incidence and preserved normal insulin secretion. In addition, MOE treatment specifically attenuated the pro-inflammatory response mediated by T helper 17 cells and enhanced anti-inflammatory T helper 2 and T regulatory cells through the impact on specific signalling pathways and transcription factors, preserved β-cells from in vitro apoptosis via blockade of caspase 3. Rosmarinic acid, exhibited only partial protection from diabetes induction.	Vujicic et al., 2015 [82]
Prebiotics	Intragastric gavage	Male Wistar rats	Animals were distributed into four groups: control (CTL); control Kefir (CTLK); diabetic (DM) and diabetic Kefir (DMK). Starting on the 5th day of diabetes, Kefir was administered by daily gavage at a dose of 1.8 mL/day for 8 weeks.	DMK rats showed a significant reduction of iNOS expression. Moreover, the DMK group presented a significant reduction of glycogen accumulation within the renal tubules when compared to the DM group.	Punaro et al., 2014 [83]
Zinc	Intragastric gavage	Wistar albino male rats	Animals were divided into four groups: control group, group supplemented with 30 mg/kg/day zinc as zinc sulfat;The third group: treated with streptozotocin to induce diabetes; fourth group: treated with streptozotocin (STZ) and supplemented with zinc. The experiments were carried out over a 6-weeks period starting 3 days after STZ injection.	Zinc supplementation showed a protective effect against diabetic damage of kidney tissue through stimulation of metallothionein synthesis and regulation of the oxidative stress. The activity of glutathione peroxidase did not change in any of the four groups.	Özcelik et al., 2012 [84]

## Supplementary Materials

The following are available online at https://www.mdpi.com/2076-3921/10/2/261/s1, Figure S1: Chemical structure of the principal exogenous antioxidants.

## Abbreviations

ALA	alpha-lipoic acid
ALT	alanine transaminase
AST	aspartate transaminase
AP-1	activator protein 1
CAMP	cathelicidin antimicrobial peptide
CAT	catalase
CCL	chemokine motif ligand
CML	carboxymethylisine
COPD	chronic obstructive pulmonary disease
CoQ	Coenzyme Q
COXs	cyclooxygenases
DAMPS	damage-associated molecular patterns
DM	diabetes mellitus
DNA	deoxyribonucleic acid
EDSS	expanded disability status scale
ENA	European Nutraceutical Association
FeNO	fractional exhaled nitric oxide
FRAP	Ferric reducing antioxidant power
FSP	fermented soy permeate
GPx	glutathione peroxidase
GSH	glutathione
H_2_O_2_	hydrogen peroxide
hBD	human beta defensin
HDL	high-density liprotein
Hs-CRP	high sensitivity-C-reactive protein
IGF	insulin-like growth factor
IL	interleukin
iNOS	inducible nitric oxide synthase
IFN	interferon
IP-10/CXCL10	induced protein-10/ C-X-C motif chemokine ligand 10
IU	international unit
LDL	low density lipoprotein
MA	microalbuminuria
MCP-1	monocyte chemoattractant protein-1
MDA	malondialdehyde
MIG	monokine induced by gamma interferon
MIP	macrophage inflammatory protein
MMP-3	matrix metalloproteinase-3.
Mn	manganese
MOE	methanolic oregano extract
MPO	myeloperoxidase
MS	multiple sclerosis
NAFLD	nonalcoholic fatty liver disease
NK	natural killer
NLRP3	NLR family pyrin domain containing 3
NO	nitric oxide
NO_2_^−^	nitrogen dioxide
NF-kB	nuclear factor kappa B
RA	rheumatoid arthritis
RANTES	Regulated upon Activation, Normal T Cell Expressed and Presumably Secreted
ROS	reactive oxygen species
SLE	systemic lupus erythematosus
SOD	superoxide dismutase
TAC	total antioxidant capacity
TAS	total antioxidative status
Th	T helper
TNF-ɑ	tumor necrosis factor-alpha
TXNIP	thioredoxin-interacting protein
VEC	vitamin E content
VEGF	vascular endothelial growth factor
WBCT	whole-body cryotherapy treatment
