# Supplementary material for: Nutraceuticals against Oxidative Stress in Autoimmune Disorders
Source: Antioxidants (Basel). 2021 Feb 8;10(2):261. doi: 10.3390/antiox10020261 (PMC7914737; doi:10.3390/antiox10020261)
Supplement: Supplementary file 1 [file antioxidants-10-00261-s001.pdf]

Supplementary Materials

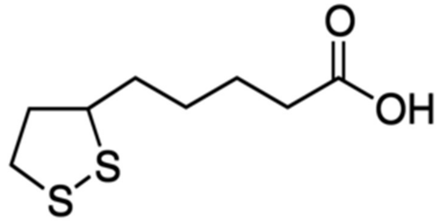

α-Lipoic acid

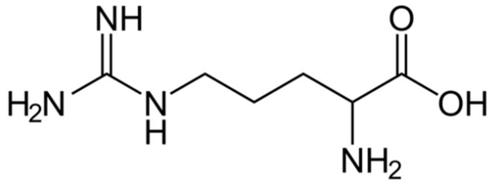

Arginine

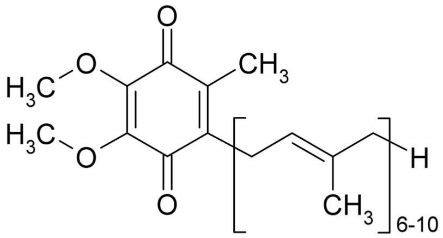

Coenzyme Q10

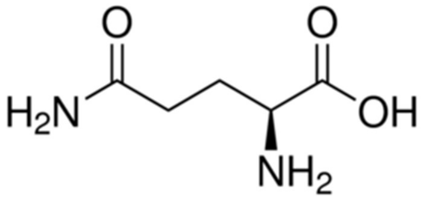

Glutamine

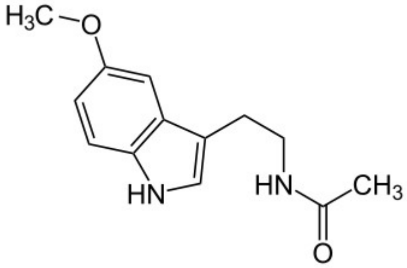

Melatonin

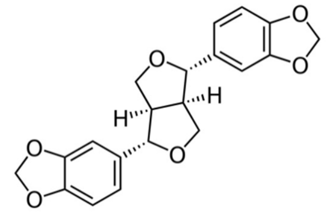

Sesamin

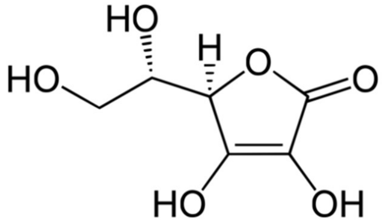

Vitamin C

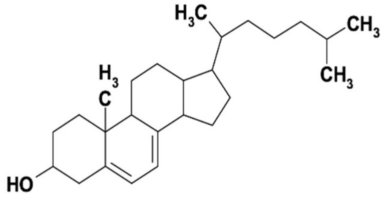

Vitamin D

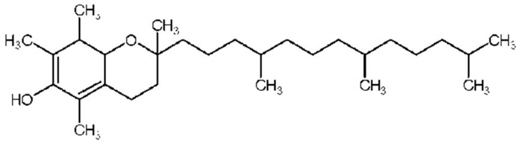

Vitamin E

FLAVONOIDI

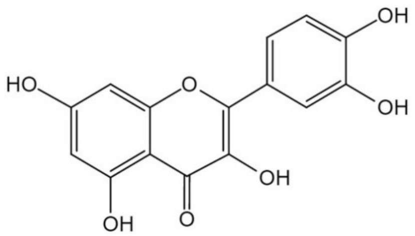

Quercetin

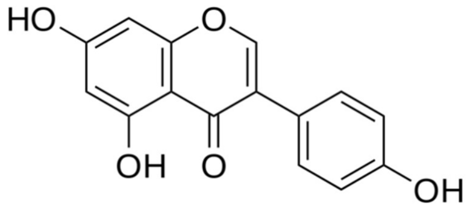

Genistein

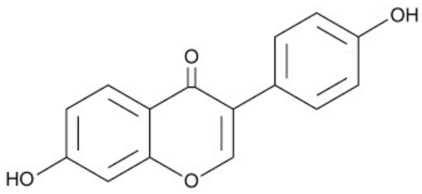

Daidzein

Figure S1. Chemical structure of the principal exogenous antioxidants.
